# Conjoining Trees for the Provision of Living Architecture in Future Cities: A Long-Term Inosculation Study

**DOI:** 10.3390/plants12061385

**Published:** 2023-03-20

**Authors:** Max D. Mylo, Ferdinand Ludwig, Mohammad A. Rahman, Qiguan Shu, Christoph Fleckenstein, Thomas Speck, Olga Speck

**Affiliations:** 1 Plant Biomechanics Group @ Botanic Garden Freiburg, Faculty of Biology, University of Freiburg, D-79104 Freiburg, Germany; 2 Cluster of Excellence *liv*MatS @ FIT—Freiburg Center for Interactive Materials and Bioinspired Technologies, University of Freiburg, D-79110 Freiburg, Germany; 3 Department of Microsystems Engineering—IMTEK, University of Freiburg, D-79110 Freiburg, Germany; 4 Green Technologies in Landscape Architecture, Research Group Baubotanik, School of Engineering and Design, Technical University of Munich, D-80333 Munich, Germany; 5 Strategic Landscape Planning and Management, School of Life Sciences, Weihenstephan, Technical University of Munich, D-85354 Freising, Germany

**Keywords:** Baubotanik, branch junction, grafting, inosculation, living architecture, micro-computed tomography, pipe model theory, urban green infrastructure, 3D reconstructions

## Abstract

Faced with the environmental challenges posed by climate change, architects are creating nature-based solutions for urban areas, such as transforming living trees into artificial architectural structures. In this study, we have analyzed stem pairs of five tree species conjoined for more than eight years by measuring the stem diameters below and above the resulting inosculation and by calculating the respective diameter ratio. Our statistical analyses reveal that *Platanus* × *hispanica* and *Salix alba* stems do not differ significantly in diameter below inosculation. However, in contrast to *P.* × *hispanica,* the diameters of the conjoined stems above inosculation differ significantly in *S. alba*. We provide a binary decision tree based on diameter comparisons above and below inosculation as a straightforward tool for identifying the likelihood of full inosculation with water exchange. Moreover, we have compared branch junctions and inosculations by means of anatomical analyses, micro-computed tomography, and 3D reconstructions showing similarities in the formation of common annual rings that increase the capacity for water exchange. Due to the highly irregular cell arrangement in the center of the inosculations, cells cannot be assigned clearly to either of the stems. In contrast, cells in the center of branch junctions can always be attributed to one of the branches.

## 1. Introduction

### 1.1. Conjoined Trees as Architectural Structures

Nature has inspired architects throughout history. Increasing urbanization, continuous changes in our landscapes and ecosystems, and the multiple challenges of climate change are leading architects and landscape designers to rely more on nature-based solutions and urban green infrastructure [1]. In light of the ecological challenges arising from climate change, the creation of living architecture by shaping and merging trees has been adopted in recent decades by architects and designers worldwide [2,3,4,5,6]. In this context, increased attention is being paid to incorporating ecosystem services for cities such as cooling, carbon storage, removal of air pollution, and reduction in rainfall runoff through a network of high-quality natural areas such as street trees, parks, and urban forests [7,8,9,10,11,12].

**Figure 1:**
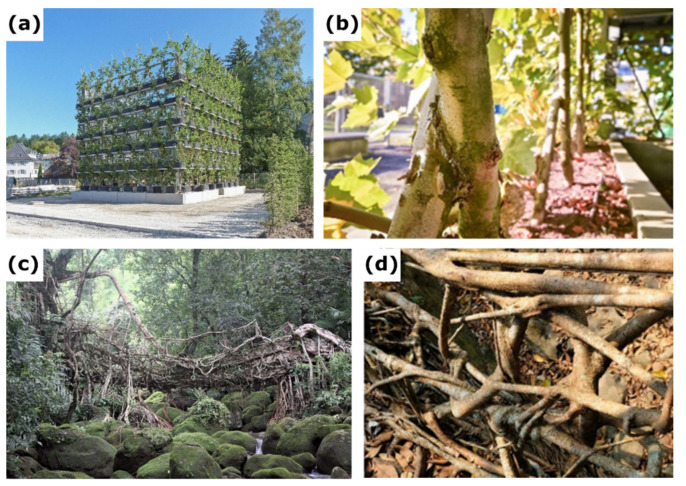
Examples of Baubotanik structures based on inosculations of stems and roots. (**a**) Three-story Baubotanik structure “Plane Tree Cube” grown in Nagold, Germany, in 2012 (photo: F. Ludwig, taken from [12]). (**b**) Detail of the “Plane Tree Cube” showing inosculations of stems of *Platanus* × *hispanica* after crosswise connection fixed with a screw (photo: F. Iannone, taken from [12]). (**c**) Living root bridge in India (reprinted under Creative Commons Attribution 4.0 International License from [17]). (**d**) Detail of a living root bridge showing inosculated roots after the individual roots have been intertwined and interwoven without additional fixation (photo: F. Ludwig, taken from [16]).

This urban network of natural areas also includes living trees that are transformed into artificial architectural structures, some of which are multi-story, while preserving their ecosystem services (Figure 1a). This approach of designing and building living architecture using living plants has been termed “Baubotanik” in the context of scientific research carried out in 2007 at the University of Stuttgart, Germany [13,14,15,16] (Figure 1). Stems, branches, or roots of trees are joined together in such a way that they merge into new physiological units with mechanically strong connections, so-called inosculations (Figure 1b,d).

The shaping of trees and the joining of trees through inosculation is based on a technique that has been used for centuries in horticulture and historical forms of building. Inosculation is a naturally occurring phenomenon when trunks, branches, or roots of two trees grow together. The term derives from the Latin word *ōsculārī*, which can be translated as “to kiss” or “to touch closely” or “to union”. For instance, living root bridges are functional load-bearing structures grown from *Ficus elastica* roots by rural Khasi and Jaintia communities in Meghalaya (India) (Figure 1c,d). Formed with no tools of contemporary engineering design, they are a unique example of vernacular living architecture [17,18]. The German Tanzlinden (“dancing lime trees”) are examples of shaping trees to create broad and accessible canopies under which social gatherings can take place [19,20,21]. In product design, approaches are increasingly being explored to “grow” chairs and other furniture on plantations by shaping and grafting trees [22]. Gentle pruning and the shaping of individual trees are established methods of arboriculture to prevent undesirable development, to maintain traffic safety, and to preserve the vitality of a tree [23,24]. When transferring these traditional approaches to the context of modern architecture, landscape architecture, and urban planning, challenges arise that require a systematic scientific basis, specific design, and engineering methods.

### 1.2. State of the Art on Inosculation Studies

However, so far, no systematic studies have been carried out on the long-term morphological and anatomical development of artificially induced tree junctions. Moreover, only initial field tests have been performed with respect to the mechanics of such junctions, and gaps remain in the comprehensive understanding of the artificial joining of branches and their long-term development [6,25]. In contrast to inosculations of tree branches and stems, those of roots have been well-investigated. Root inosculations [26] have been observed in more than 150 tree species [27] (Figure 1d), with up to 90% of a tree stand being interconnected [28]. Tarroux and DesRochers [29] have found that root inosculation is an energetically costly process, but one that is subsequently non-prejudicial and that can have a positive effect on tree growth. The use of a communal root system allows the maximum use of resources by redistributing them among the individual trees, leading to increased radial tree growth. Basnet et al. [30] have demonstrated that, through the interconnection of large roots, subterranean inosculations provide additional support and stability to trees. Bormann and Graham [31] have described and quantified the inosculation process for roots of the eastern white pine (*Pinus strobus*) based on physiological experiments with dyed water. Nevertheless, the conjoining of multiple trees anatomically might favor the spread of diseases.

Millner [32] has provided a detailed description of the inosculation process for the self-attaching stems of *Hedera helix*, which form anastomosing networks of climbing stems that supply water and nutrient salts to the upper stems from distant stems. According to Millner [32], the inosculation process of ivy stems is preceded by a number of developmental steps that result directly from pressure at the point of contact. Bark tissues are compressed at the contact site and are deflected outward as the fusion between the stems becomes more complete. As a result, the dead outer bark tissues rupture in the marginal areas, and a wound periderm is formed. During increasing wound-induced growth, the two healing calluses unite with one another, resulting in increasing connections and the transfer of water and nutrients from one stem to the other [32,33]. Thus, grafting can result in tissue fusion, vascular continuity, and finally, a single physiologically and mechanically functioning unit [33].

Inspired by this natural inosculation process, the Research Group Baubotanik (recently set up at the Technical University of Munich, Germany) has examined various techniques for the initiation of artificial inosculations and has investigated their applicability for a variety of tree species in the construction of living tree structures. From 2008–09, Ludwig established an initial series of test plantings to investigate various approaches for the initiation of inosculations by pressing shoots firmly onto each other. The stems were connected at one point either by crossing (crosswise connection) or bending (tangential connection) using either thin ropes or screws for fixation. Unlike horticultural grafting, the bark tissues were not cut in order to place the cambium of one plant directly upon that of another. Therefore, Ludwig [15] could test and develop techniques that were straightforward, comparably fast, cheap, labor-saving in implementation, and applicable under construction site conditions.

### 1.3. Inosculations Form Functional Units

**Figure 2:**
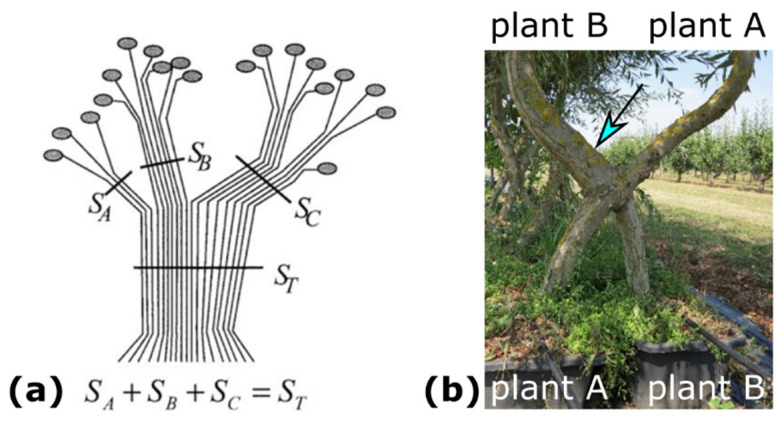
Branching and grafting of trees. (**a**) Plant modeling based on the pipe model theory and representing a branching system (reprinted with permission from [37]).The number of unit pipes in the trunk (*S_T_*) is equal to the sum of unit pipes of the three branches (*S_A_* + *S_B_* + *S_C_*) connected above it [38]. (**b**) Artificially conjoined *Salix alba* plants planted in two separate pots and connected in a crosswise fashion. The stem diameter of Plant A above inosculation (blue arrow) is markedly larger than its stem diameter below inosculation.

The relationship between morphometric variables (e.g., stem diameters below and above the inosculation) and physiological unity (e.g., water supply) of individual Plants A and B being joined is considered in the pipe model theory [34,35,36,37,38]. Shinozaki et al. [34,35] have proposed an interpretation of the observed linear relationship between the amount of stem tissue and the correspondingly supported leaves. In the pipe model for trees, each pipe represents a vascular tube providing water from the roots to the leaves, with the amount of stem tissue being measured in terms of stem diameter or sapwood area. In Figure 2a, a tree stem is represented as a bundle of unit pipes, each pipe supplying water to a unit of leaves; the number of unit pipes in the tree trunk is equal to the total number of unit pipes that compose the branches connected above it [37,38]. Figure 2b shows two trees, each in its own pot, after crosswise connection and the formation of an inosculation. The stem diameter below the inosculation of Plant B is markedly smaller than that of Plant A. Interestingly, above the inosculation, exactly the opposite is seen, with the stem diameter of Plant B being clearly larger (see arrow in Figure 2b) than that of Plant A. In Plant B, the stem diameter above inosculation is markedly larger than the stem diameter below inosculation. This differs from the appearance of non-inosculated individual trees, which taper towards their tips and, thus, have increasingly smaller diameters toward the apex [39,40].

We, therefore, hypothesize that a physiologically functional inosculation has developed between Plant A and Plant B, and that the originally separate root and crown areas are interconnected, allowing water to pass from one former individual stem to the other. On the basis of the pipe model theory [34,35], we assume the following: girth growth will respond to the resulting changes in transport requirements, and trunk sections with more water flow will produce more water-conducting sapwood and, therefore, will grow more in girth [34,35,36,37,38]. We also hypothesize that inosculated stems with an asymmetric crown and root development should, on average, show a ratio between the stem diameter above and below the inosculation (“taper”) differing from the ratio of non-inosculated individual trees. Thus, if the tree pairs studied show this divergent diameter development, we can assume that water is transported across initial individual boundaries, and that the two trees act (at least partly) as one physiological unit. Based on these assumptions and related comparative studies carried out on various tree species, our present work aims to test our hypothesis that inosculations form not only a structural unit, but also a physiological unit with respect to water conductivity. In the practice, this would mean that in conjoined trees, at best, water exchange can only be assumed based on measurable morphological differences between the stems. Thus, one stem below the inosculation could be removed because both stems above the inosculation will be sufficiently supplied with water by the remaining stem (compare the technique of “plant addition” as described in [2]).

### 1.4. Aim of the Study

In the context of this study, our goal was to find answers to two scientific questions. In terms of practical issues of Baubotanik, we address the question: “Are simple morphometric measurements suitable for assessing whether interconnected stems have merged into a physiological unit with respect to water exchange?” In the context of comparative analyses, we sought answers to the question: “What similarities and differences characterize artificial inosculations and natural branch junctions?” With a focus on *Platanus* × *hispanica* and *Salix alba*, we, therefore, investigated conjoined tree samples with respect to morphometric properties such as stem diameters below and above the inosculation and the ratios thereof. Furthermore, we compared inosculations and branch junctions with respect to anatomical properties derived from serial sections and micro-computed tomography (µ-CT). Moreover, we created volumetric 3D reconstructions of the macroscopic structure with a focus on the development of common growth rings based on serial sections in which water-conducting tissues have been stained with a magenta ink. Based on our results on measurable morphometric criteria, we present a binary decision tree as a straightforward tool for identifying the likelihood of complete inosculation with water exchange.

## 2. Material and Methods

### 2.1. Plant Material

All conjoined stem pairs originated from test fields set up from 2008–09 [15] and 2011 [41]. To initiate inosculations, the shoots of 1-to-3-year-old plants were connected at one point along the stems either by the crossing (crosswise connection) or bending (tangential connection) of the stems, with either thin ropes or screws being used for fixation. Detailed information about the tree species, their types of connection (crosswise or bending), fixation (rope or screw), and height of inosculation can be found in Supplementary Materials Table S1.

### 2.2. Morphometric Studies

**Figure 3:**
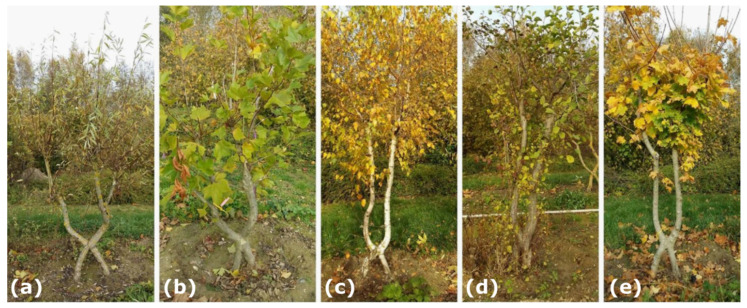
Typical examples of the five tree species with inosculated stems after crosswise connection that are included in the morphometric investigations. (**a**) *Salix alba*; (**b**) *Platanus* × *hispanica*; (**c**) *Betula pendula*; (**d**) *Alnus glutinosa*; and (**e**) *Acer platanoides*.

In autumn 2019, we performed morphometric investigations of 32 inosculation samples, including 17 samples of *Salix alba* L., eight samples of *Platanus* × *hispanica* auct. non Mill. ex Münchh., nom. dub (a hybrid of *Platanus orientalis* and *Platanus occidentalis*), two samples of *Acer platanoides* L., four samples of *Betula pendula* Roth, and one sample of *Alnus glutinosa* (L.) Gaertn. (Figure 3). Of the 32 samples, 26 were connected by the crosswise arrangement and six by the bending of the stem pairs. In addition, 27 samples were fixed with a rope and five with a screw. Raw data of the diameters of the plant stems above and below the inosculation are provided in Supplementary Materials Table S1.

We measured the diameters of Plant Stems A and B, both about 100 mm above ground surface (*b*) and 350 mm above the inosculation (*t*). Diameters were measured in two perpendicular directions by using a digital caliper, and the arithmetic mean values were calculated. Based on these mean values of *At*, *Ab*, *Bt*, and *Bb*, we calculated the dimensionless diameter ratios (*DR,* “taper”) of Plants A (*At*/*Ab*) and B (*Bt*/*Bb*), and the taper ratio (*TR*) of the conjoined sample by using the following equations:

*DR*_*A* [-] = (*At*/*Ab*) (1)

*DR*_*B* [-] = (*Bt*/*Bb*) (2)

*TR* [-] = (*At*/*Ab*)/(*Bt*/*Bb*) (3)

**Figure 4:**
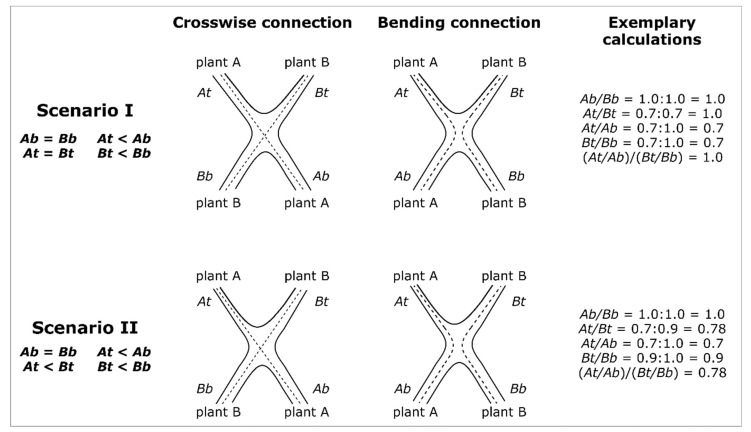
Schematic representation of possible diameter developments after inosculation of crosswise and bending connection of Plant A and Plant B. In order to distinguish between Plants A and B, we define the following: *At*/*Ab* ≤ *Bt*/*Bb.* In Scenarios I and II, the diameters below the inosculation are equal (*Ab* = *Bb)*, and the diameters above the inosculation are markedly smaller than those below, resulting in a taper (*At* < *Ab* and *Bt* < *Bb*). In Scenario I, the diameters above inosculation are the same (*At* = *Bt*), which infers an equal development of Plants A and B. Therefore, the diameter ratios of A and B have the same value (*DR_A* = *DR_B*) and, thus, the taper ratio is *TR* = 1.0 (see exemplary calculation). However, in Scenario II, the plants develop unequally, reflected by smaller diameters of Plant A compared with those of Plant B above the inosculation (*At* < *Bt*). Therefore, the diameter ratios of A and B have different values (*DR_A* < *DR_B*), and the taper ratio is *TR* < 1.0 (see exemplary calculation).

The Plant Stems A and B are defined in such a way that *At*/*Ab* is smaller than *Bt*/*Bb*, which implies that *TR* is in the range between 1 and 0. Figure 4 presents two scenarios with exemplary calculations that apply to crosswise and bending connections. If the diameters of Plant A and Plant B above and below the inosculation are equal, their taper ratio *TR* = 1.0 (Scenario I). On the contrary, *TR* < 1.0 if the diameters of Plant A and Plant B below the inosculation are equal, and if the diameter above the inosculation differs in such a way that *At* < *Bt* (Scenario II). In both scenarios, a taper of the individual stems is assumed (*At* < *Ab* and *Bt* < *Bb*).

### 2.3. Macroscopic Anatomical Studies and 3D Reconstruction

Anatomical studies were carried out on one sample of *S. alba*, which was conjoined by bending connection and rope fixation (sample #18, see Supplementary Materials Table S1), and on one sample of *P.* × *hispanica*, which was conjoined by crosswise connection and screw fixation (sample #33, see Supplementary Materials Table S1). The samples were cut into horizontal slices with a thickness of 5 mm by using a band saw. After each cut, the exposed surfaces were scanned with a flatbed scanner (Kyocera TASKalfa 2552ci KX, Kyōto, Japan) in life size. By spacing the cuts equally and fixing them to a wooden plate, each cut was assigned a distinct position in a three-dimensional (3D) coordinate system.

For each scan, the growth rings were manually redrawn in a CAD program (AutoCAD 2020, Autodesk, San Francisco, CA, USA) as a closed polyline. Subsequently, these 2D transverse digital drawings were arranged according to their spatial positions in 3D space. Non-uniform rational B-Splines (NURBS) surfaces were then computed between corresponding growth rings in adjacent sections by using the “sweep” command in Rhino 5 (Robert McNeel & Associates, Seattle, WA, USA). At points at which two growth rings merged into one, a spatial quadrilateral was added for a smooth connection. In this way, a 3D model was created to illustrate layers of the continuous growth surfaces, enabling an observation of the inosculation samples from any perspective. Bark tissue was not incorporated in the model creation.

For each scan, the wood tissue of the two stems after the formation of the first common growth ring was marked, and the percentage area *A(com),* with respect to the total area, was calculated.

### 2.4. Micro-CT Scans

We investigated comparable structures of branch junctions and inosculations in greater detail with micro-computed tomography (µ-CT). We harvested one sample of *S. alba* with inosculation after crosswise connection and rope fixation (sample #25, see Supplementary Materials Table S1), and one branch junction of the same plant pair. Moreover, we harvested one sample of *P.* × *hispanica* with inosculation after crosswise connection and screw fixation (sample #14, see Supplementary Materials Table S1) and one branch junction of the same plant pair. The inosculations and the branch junctions were cut into cubes of approximately 20 mm of edge length by using a circular saw. For the µ-CT scan, one cube per sample was selected that best represented the potentially corresponding tissue areas identified in the macroscopic examinations. All samples were acquired as 360° scans at 10 µm resolution by using the SkyScan1272 scanner with a source voltage of 60 kV, a source current of 166 µA, a 0.25 mm Al filter, 0.3° rotation steps, and SkyScan software (version 1.1.10; both Bruker Corporation, Billerica, MA, USA). NRecon software (version 1.6.10.1, Micro Photonics Inc., Allentown, PA, USA) was used for data reconstruction, for application of the ring artefact and beam hardening correction, and for smoothing of the data (over five pixels; only for the inosculation sample of *P.* × *hispanica*). CTvox software (version 3.3.0r1403, Bruker Corporation, Billerica, MA, USA) was used to extract videos from the reconstructed data. Avizo software (version 2020.2, Thermo Fisher, Waltham, MA, USA) was employed to extract single sections.

### 2.5. Staining of Water-Conducting Tissues

Water flow experiments were carried out with the samples described in Section 2.3 (natural branch junctions and artificial inosculations of *S. alba* and *P.* × *hispanica*). In this context, the samples were cut and oriented upside down, and a mixture of water and a few drops of magenta ink (refill ink for Canon printers) were passed by gravity from the inosculation through the two stems or from the branch junction through the two branches. The magenta ink stains the water-conducting tissue, which can be identified with the naked eye, for example, in serial cross-sections.

### 2.6. Statistics 

Morphometric raw data and descriptive statistics were recorded and analyzed with Excel (version 2016, Microsoft Corporation, Redmond, WA, USA). All data are described using the median with the respective interquartile ranges (IQR). Further statistical analyses were performed using the statistical software GNU R v.4.0.4 [42] to determine significant differences. After being tested for normal distribution (Shapiro–Wilk test) and homogeneity of variances (Levene’s test), data were subjected to *t*-tests performed with an alpha level of 0.05. Statistical significance is indicated by *p* < 0.05 (*), *p* < 0.01 (**), and *p* < 0.001 (***). Raw data are provided in the Supplementary Materials Table S1, and statistical analyses of *P.* × *hispanica* and *S. alba* samples are given in Supplementary Materials File S1.

## 3. Results

### 3.1. Morphometric Analyses

**Figure 5:**
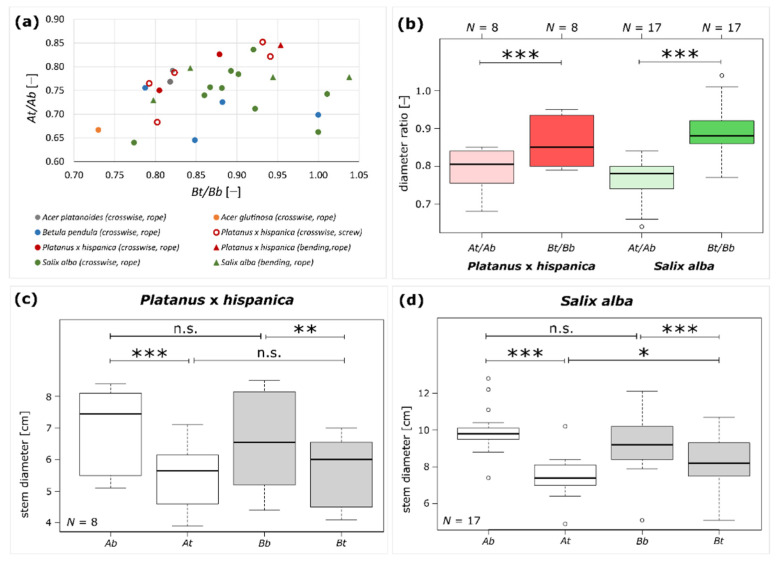
Results of morphometric analyses for long-term studied inosculated stem pairs. (**a**) Scatter plot displaying the diameter ratio *At/Ab* as a function of *Bt/Bb* for all samples. The respective species are color-coded, with *Salix alba* marked in green (*N* = 17), *Platanus* x *hispanica* in red (*N* = 8), *Betula pendula* in blue (*N* = 4), *Acer platanoides* (*N* = 2) in gray, and *Alnus glutinosa* (*N* = 1) in orange. Crosswise connection (*N* = 26) is represented by circles and bending connection (*N* = 6) is represented by triangles. Fixation by rope (*N* = 27) is indicated by filled markers, and fixation by a screw is indicated by empty markers (*N* = 5). (**b**) Boxplots of the diameter ratios *At/Ab* and *Bt/Bb* showing significant differences for both *Platanus* × *hispanica* (*N* = 8) and *Salix alba* (*N* = 17). Boxplots of measured diameters above (*t*) and below (*b*) the inosculation of Plant A and Plant B for (**c**) *Platanus* × *hispanica* and (**d**) *Salix alba*. Statistical significance is indicated by *p* < 0.05 (*), *p* < 0.01 (**), and *p* < 0.001 (***).

The diameters of Plant A and Plant B were measured above (*t*) and below (*b*) the inosculation of 33 samples from five species. The diameter ratio of Plant A (*At/Ab*) and Plant B (*Bt/Bb*) and the taper ratio *TR* of each sample was calculated from these data according to Equations (1)–(3). In Figure 5a, we compiled all available data into one chart that plots *At*/*Ab* as a function of *Bt*/*Bb*. Since some of the sample sizes are very small, it was not possible to perform statistical tests. However, a qualitative statement can nevertheless be made, namely, that no clustering was revealed with respect to the tree species, the type of connections (crosswise or bending), or the fixations (rope or screw).

Further statistical tests were limited to *P.* × *hispanica* and *S. alba*, as sufficient sample sizes were only available for these tree species (Figure 5b–d). Since we found no significant differences in *P.* × *hispanica* for the diameters *At, Ab*, *Bt, Bb*, diameter ratios *At/Ab* and *Bt/Bb,* and the taper ratio *TR* between fixation with a rope or a screw, we pooled all data (all unpaired *t*-tests: *p* > 0.05). We also pooled the data from *S. alba* because we found no significant differences in the diameters *At, Ab*, *Bt, Bb*, diameter ratios *At/Ab* and *Bt/Bb,* and the taper ratio *TR* between bending and crosswise connections (all unpaired *t*-tests: *p* > 0.05). The median and interquartile range of the measured and calculated variables carried out for *P.* × *hispanica* and *S. alba* are given in Table 1.

For *P*. × *hispanica*, no significant differences are found when comparing the diameters *At* and *Bt* or *Ab* and *Bb* (all paired *t*-tests: *p* > 0.05). However, the diameters above the inosculation are significantly smaller than those below the inosculation for both Plant A (paired *t*-test: *p* < 0.001) and Plant B (paired *t*-test: *p* < 0.01) (Figure 5c). Figure 5b shows that the diameter ratio (*Bt/Bb*) of Plant B is significantly higher than the diameter ratio of Plant A (*At/Ab*) (paired *t*-tests: *p* < 0.001). For *S. alba*, the diameters *At* are significantly smaller than *Bt* (paired *t*-test: *p* = 0.03), whereas *Ab* and *Bb* do not differ significantly (paired *t*-test: *p* > 0.05). Moreover, the diameters above the inosculation are significantly smaller than those below the inosculation for both Plants A and B (paired *t*-test: *p* < 0.001) (Figure 5d). Figure 5b shows that, for *S. alba*, the diameter ratio (*Bt/Bb*) of Plant B is significantly higher than the diameter ratio of Plant A (*At/Ab*) (paired *t*-tests: *p* < 0.001). The taper ratio *TR* of *P.* × *hispanica* ranges between 0.85 and 0.97 and of *S. alba* between 0.66 and 0.96. Raw data and statistical analyses are given in Supplementary Materials Table S1 and File S1, respectively.

### 3.2. Macroscopic Anatomical Analyses

**Figure 6:**
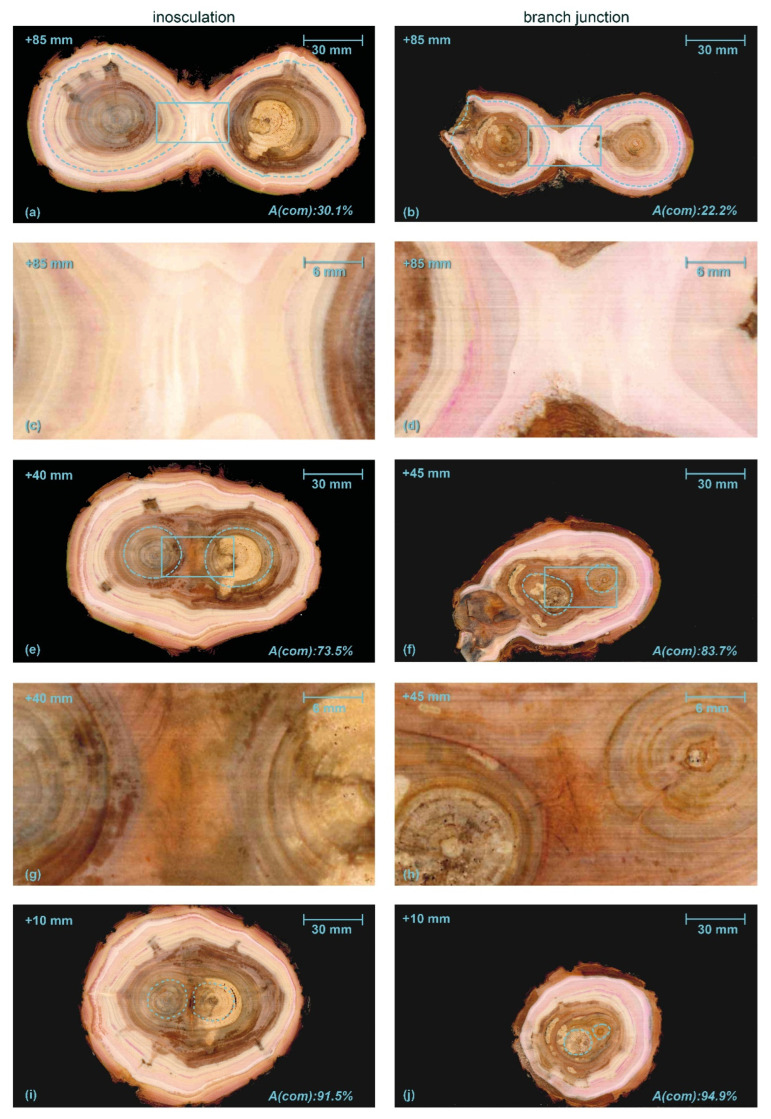
Serial sections through an inosculation after bending connection and rope fixation (Sample #18, **left**) and a branch junction (**right**) of *Salix alba*. *A(com)* is the percentage of wood area grown after the formation of the first common growth ring (common wood tissue) of the two branches with respect to the total area. In both samples, we find brownish heartwood and signs of decay in the central area. Dashed light blue lines in (**a**,**b**,**e**,**f**,**i**,**j**) mark the largest individual growth rings, solid blue lines in (**a**,**b**) and (**e**,**f**) highlight the central areas shown in detail in (**c**,**d**) and (**g**,**h**), respectively. The millimeter specification top left in each image refers to the apical position relative to the center of inosculation and branching. The magenta coloring of the water-conducting tissues results from previously performed water flow experiments with a mixture of water and magenta ink.

Figure 6 shows typical serial transverse sections of *S. alba* comparing an inosculation after bending connection and rope fixation (sample #18) and a natural branch junction. The Video S1 (Supplementary Materials) includes transverse sections, starting at the point of inosculation or branch junction and proceeding upward in 5 mm intervals. Whereas two stem centers are visible from the beginning onward in the inosculation, various side shoots are found next to the main shoots (15 mm and 45 mm sections) in the natural branch junction. Finally, the main shoot is no longer present from the 70 mm section onward.

Figure 6a,b shows a section overview about 85 mm above the center of the inosculation and the branch junction, respectively. The dashed light blue lines mark the largest growth ring in which no tissue connections are visible. The tissues outside these lines are merged in the middle region and subsequently form a common growth ring. The common wood tissues and the outer growth rings of the unconnected tissues (especially in the natural branch) are lightly colored by magenta ink from previous water flow experiments.

The percentage of cross-sectional area that is common wood tissue (*A*(*com*), in relation to the total cross-sectional area of the wood) is shown at the bottom right in each image. The central areas of each section highlighted by the rectangles with solid light blue line in Figure 6a,b are shown in detail in Figure 6c,d, respectively. In both cases, the central area consists of relatively homogeneous wood tissue without inclusions of other tissue types. Figure 6e,f show the sections approximately 40 or 45 mm above the points of inosculation and branching, respectively, where the central area (Figure 6g,h) is characterized by outwardly deflected tissue directions. At the center of both samples, wound tissue and small inclusions are visible; these are presumably compressed remnants of the bark tissue. This is slightly more pronounced in the branch junction than in the inosculation. Magenta staining, which documents water transport, is found in the periphery at the outer three-to-four common growth rings. The magenta staining is more pronounced in the natural branch sample. Figure 6i,j show the situation slightly (10 mm) above the inosculation or branching point, where, in the inosculation, enclosed bark tissues are present. In both samples, the magenta-stained water-conducting tissues form a common ring in the periphery.

**Figure 7:**
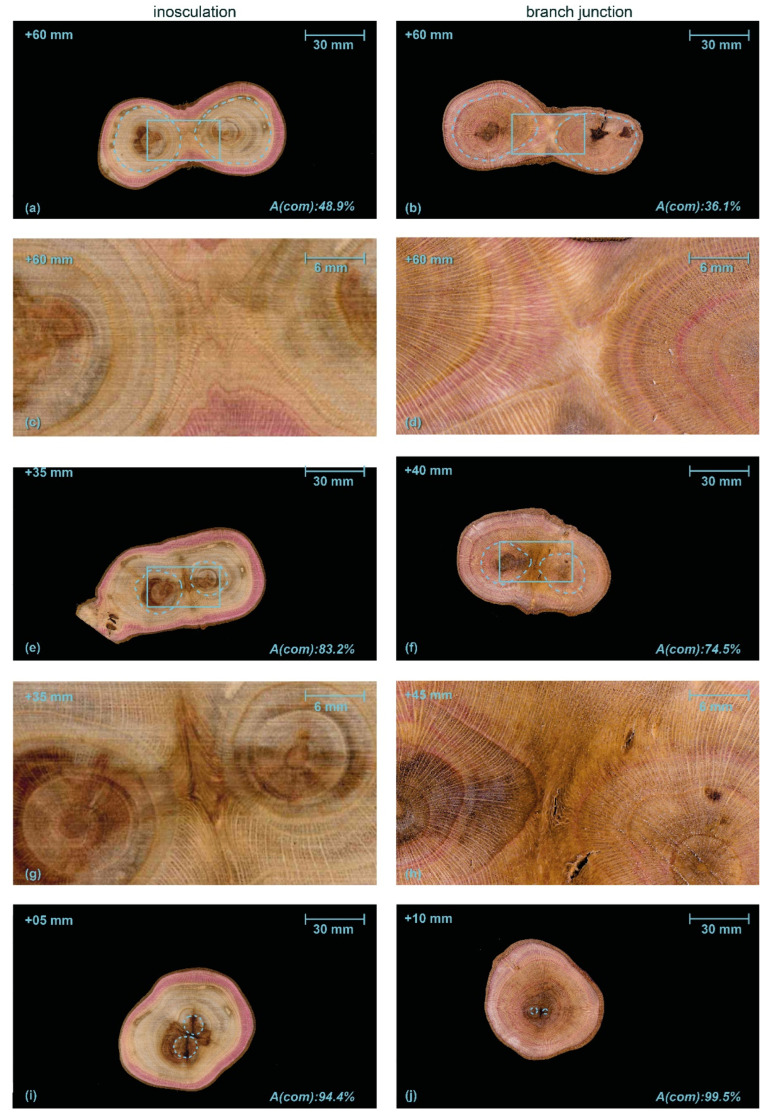
Serial sections through an inosculation after crosswise connection (**left**) and a branch junction (**right**) of *Planatus* x *hispanica* (sample #33). *A*(*com*) is the percentage of wood area grown after the formation of the first common growth ring (common wood tissue) of the two branches with respect to the total area. In both samples, we find brownish heartwood and signs of decay in the central area. Dashed light blue lines in (**a,b,e,f,i,j**) mark the largest individual growth ring, solid blue lines in (**a**,**b**) and (**e**,**f**) highlight the central areas shown in detail in (**c**,**d**) and (**g**,**h**), respectively. The millimeter specification top left in each image refers to the apical position relative to the center of inosculation and branching. The magenta coloring of the water-conducting tissues results from previously performed water flow experiments with a mixture of water and magenta ink.

Figure 7 presents typical serial transverse sections of *P.* × *hispanica* comparing an inosculation after crosswise connection and screw fixation (sample #33) and a natural branch junction. As described for Video S1, the Video S2 (Supplementary Materials) provides transverse sections starting at the point of inosculation or branch junction and proceeding upward in 5 mm intervals. In the first three sections of the inosculation (+5, +10, +15 mm), clear but gradually decreasing, slightly offset, dark brown linear discolorations are visible, with their orientation corresponding to the screw located below (Figure 7i). In the area between +5 and +20 mm, ingrown bark is clearly visible between the two shoots, whereas further above, it is no longer visible or only to a much lesser extent. Higher up (from section +40 onwards), the cross-sections are characterized by ingrown branch stubs and discoloration originating from them; other than this, the wood appears homogeneous (Figure 7a,c). In all inosculation sections, the outer two-to-three annual rings are distinctly colored magenta, whereas the rest of the wood shows no magenta coloration. Particularly in the area of the joint, the inner two annual rings, especially of the left plant, are discolored brown. In the branch junction, the two stem centers are visible even in the first sections (Figure 7j). In the sections at +10 mm and +15 mm, ingrown bark is visible to a small extent. Darkly discolored ingrown branch stubs or something similar also appear further up (from +55 mm on; Figure 7b,d). Almost all annual rings are stained magenta.

### 3.3. Volumetric 3D Reconstruction

Based on 3D reconstructions of the growth rings of *S. alba* and *P.* × *hispanica*, we reconstructed the development of the wood volume from an inosculation and a natural branch junction. In this 3D reconstruction, we considered the growth rings over a period of eight or ten years, depicting them as semi-transparent light gray surfaces with the first common annual growth ring marked in blue. The pith is marked in dark gray.

**Figure 8:**
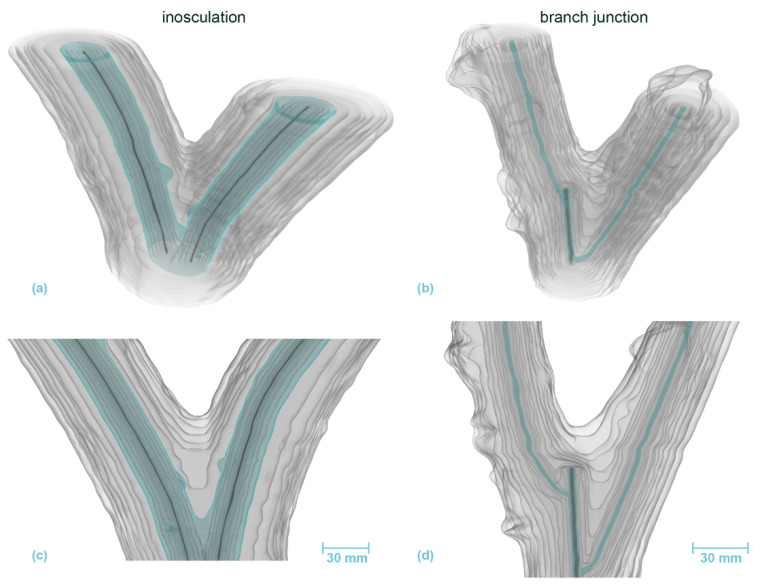
Volumetric 3D reconstruction of an inosculation after bending connection (**left**, sample #18) and a branch junction (**right**) of *Salix alba*. (**a**,**b**) are perspective views, (**c**,**d**) are longitudinal sections in front view. The growth rings are indicated in gray, with the first common growth ring highlighted in blue. The pith is shown in dark gray.

**Figure 9:**
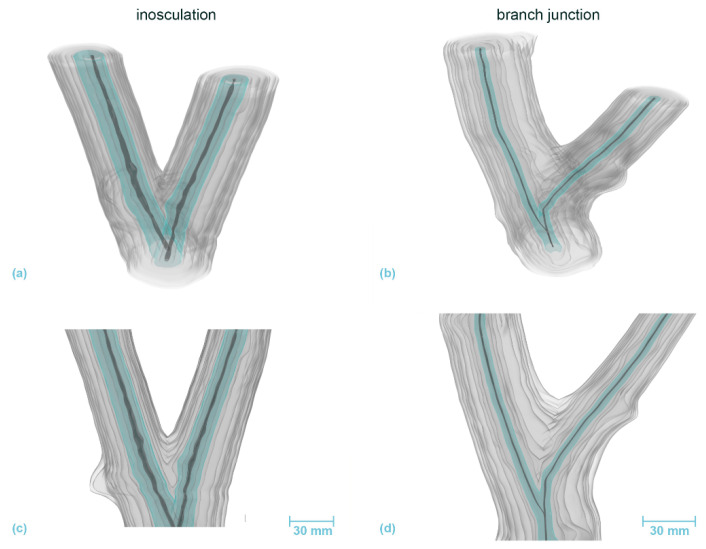
Volumetric 3D reconstruction of an inosculation after crosswise connection (**left**) and a branch junction (**right**) of *Planatus* × *hispanica*. (**a**,**b**) are perspective views, (**c**,**d**) are longitudinal sections in front view. The growth rings are indicated in gray, with the first common growth ring highlighted in blue. The pith is shown in dark gray.

Figure 8 and Figure 9 illustrate the 3D-reconstructed inosculated samples (left column) and the natural branch junction (right column) of *S. alba* and *P.* × *hispanica*, respectively. In the inosculation of *S. alba*, no change can be seen in the annual ring geometry at the point of connection until the fourth year of growth. The first common annual ring appears during the sixth year of growth. In the case of the branch junction, the bifurcation develops from two side shoots that sprout out at a distance of about 30 mm apart in the second growth year. The main shoot probably died or was cut off about 10 mm above the second side shoot in the first year. Videos S3 and S4 (Supplementary Materials) show the volumetric 3D reconstruction of the inosculation and the natural branch junction of *S. alba*, respectively.

In the inosculation of *P.* × *hispanica*, the growth rings of the second year show the first signs of merged xylem tissue (Figure 9 left, marked in blue). In the case of the branch junction, two branches have emerged even during the first year. The left branch is a side shoot, the right one is either the main shoot bending to the right or another side shoot, in which case the main shoot must have died immediately after the side shoot emerged. Videos S5 and S6 (Supplementary Materials) show the volumetric 3D reconstruction of the inosculation and the natural branch junction of *P.* × *hispanica*, respectively.

### 3.4. Micro-CT Analyses

**Figure 10:**
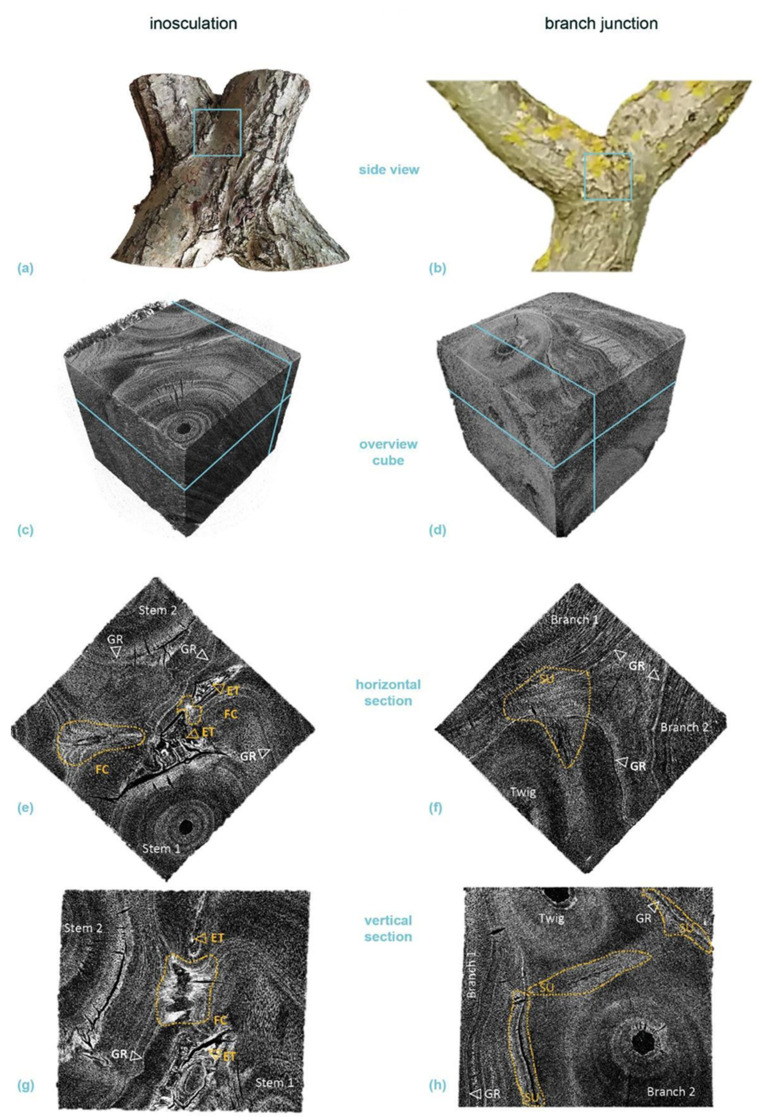
Micro-computed tomography (µ-CT) scans of a selected cube (edge length about 20 mm each) from the center of a crosswise inosculation of two stems (**left**) and a natural branch junction (**right**) of *Salix alba* (sample #25). (**a**,**b**) show images of the two original samples and the location of the cubes cut for the µ-CT scan (light blue square). (**c**,**d**) illustrate an overview scan of the cube, with the respective locations of the presented horizontal (**e**,**f**) and vertical (**g**,**h**) sections marked in light blue. The involved branches, side-twigs, growth rings (GR), fusion callus (FC), enclosed tissue (ET), and stress-induced undulations (SU, yellow) are marked in the sections.

**Figure 11:**
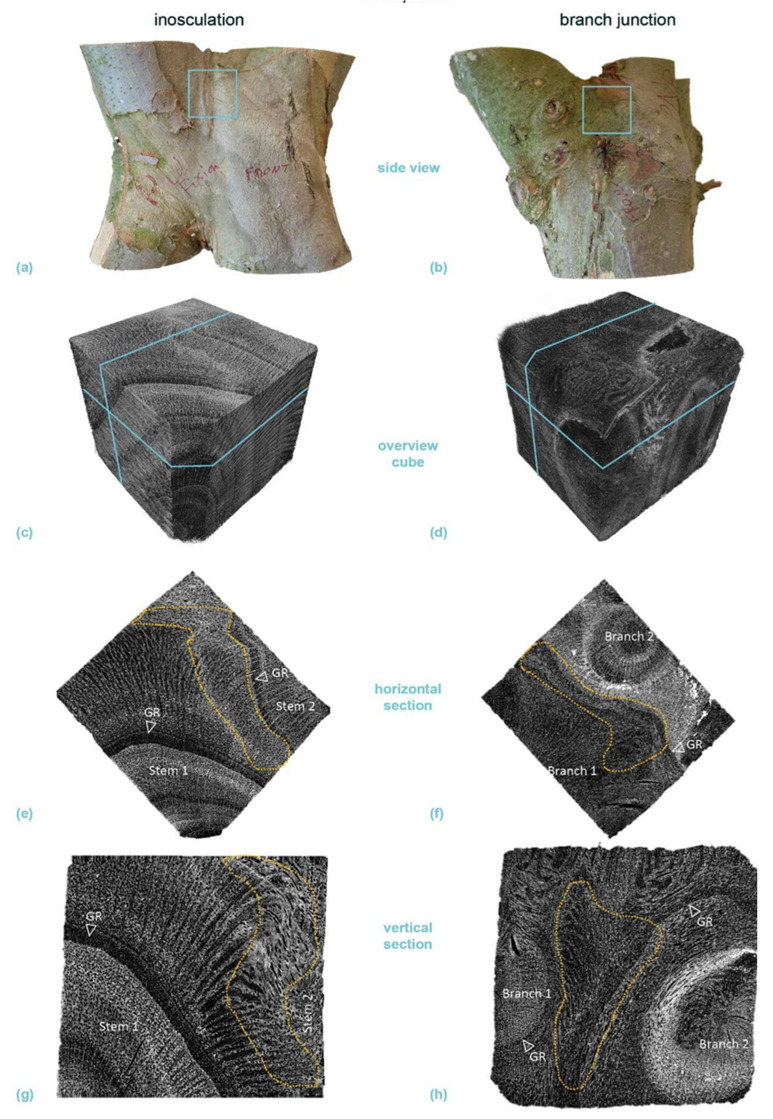
Micro-computed tomography (µ-CT) scans of a selected cube (edge length about 20 mm each) from the center of a crosswise inosculation of two stems (**left**) and a natural branch junction (**right**) of *Platanus* × *hispanica* (sample #14). (**a**,**b**) show images of the two original samples and the location of the cubes cut for the µ-CT scan (light blue square). (**c**,**d**) illustrate an overview scan of the cube, with the respective locations of the presented horizontal (**e**,**f**) and vertical (**g**,**h**) sections marked in light blue. The involved branches, growth rings (GR), and stress-induced undulations (SU, yellow) are marked in the sections.

Figure 10 and Figure 11 present the results of the µ-CT investigations comparing crosswise inosculations and natural branch junctions of *S. alba* and *P.* × *hispanica*, respectively. The reconstructed µ-CT images show the arrangement and distribution of the wood vessels at the cellular level and, for *P.* × *hispanica*, the parenchymatous tissue of the wood rays. The spatial visualization was most favorable when the cells were aligned parallel to one of the coordinate axes of the scan. In addition to the annual ring structure, stress-induced undulations could be found in all four samples (Figure 10c–f and Figure 11c–f). Additionally, the semi-ring-porous (*S. alba*) and the diffuse- to semi-ring-porous (*P.* × *hispanica*) structures of the wood were discernible.

Areas of extremely high cell density were found in the branching zones of the natural branch junctions. These connecting regions were found in the boundary zone between the tissues of the individual branches, together with irregular wood growth. However, in these areas, the cells grew alongside each other and could always be assigned to one of the two branches with no fusion callus visible (Figure 10f,h and Figure 11f,h). The cells of the two branches essentially grew past each other and could always be assigned to one of the two branches. In the inosculations, the micrographs revealed areas with highly irregular cell arrangements (Figure 10e,g and Figure 11e,g). In these areas, cells of both former individuals grew together, sometimes resulting in a slanted arrangement in relation to the actual direction of growth. A dividing line between the two stems was not always recognizable, and some areas could not be clearly assigned to one of the two individual stems (Videos S7a–d, Supplementary Materials). In *S. alba*, some of these areas were over 7 mm in size, and ordered cell arrangements were barely visible. In addition, cell fusion calluses and enclosed tissues were found between the two adjacent stems. In *P.* × *hispanica*, these areas extended over about 4–5 mm with diffuse transitions. As an overview of the µ-CT scans, we provide videos of *S. alba* showing the inosculation (Videos S7a,b, Supplementary Materials) and the natural branch junction (Videos S7c,d, Supplementary Materials) plus scans of *P.* × *hispanica* showing the inosculation (Videos S8a,b, Supplementary Materials) and the natural branch junction (Videos S8c,d, Supplementary Materials).

## 4. Discussion

**Figure 12:**
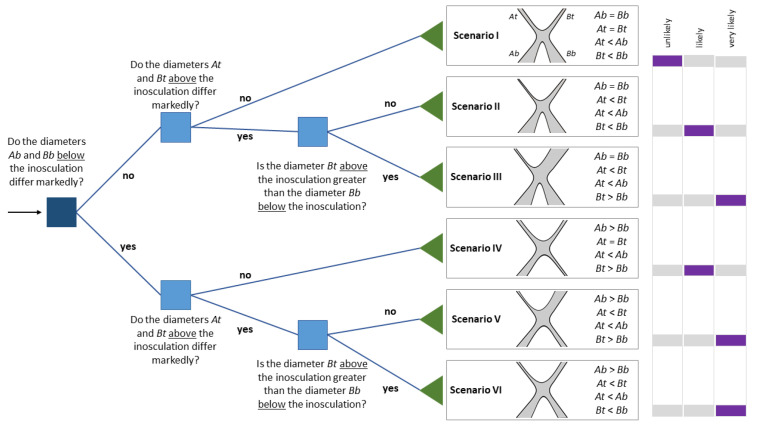
Binary decision tree as a straightforward tool for identifying the likelihood of complete inosculation with water exchange. Root node is in navy blue, internal nodes are in blue, and leaf nodes are in green. Based on an inosculation of Plant A and Plant B after crosswise or bending connection, we present six theoretical scenarios of their stem diameters below (*Ab* and *Bb*) and above (*At* and *Bt*) the inosculation (all assume a taper in Plant A, denoted as *At* < *Ab*). We set Plant A according to *At*/*Ab* ≤ *Bt*/*Bb.* The schematic drawings represent a bending connection of the tree stems. In Scenario I, the taper ratio is *TR* = 1.0; in all other scenarios, *TR* < 1.0. Complete inosculation with water exchange across individual boundaries of the two plants is more likely, the more the stem diameters differ above inosculation (*At* < *Bt*) and the more the stem diameter of Plant B is larger above than that below inosculation (*Bt* > *Bb*). It is even more likely with a combination of the two diameter developments, together with equal diameters below inosculation (*Ab = Bb*) (Scenario III). The Likert scale on the right-hand side provides a qualitative likelihood assessment for complete inosculation in each scenario, indicated by the purple bars.

We present preliminary results of a long-term study of inosculated tree stems that can be used for future living architecture applications. The objective of this study has been to find measurable morphometric and anatomical criteria that can provide an indication of whether the two initially individual tree stems have fused to the extent of mutual water exchange between Plant A and Plant B in the inosculation. Why is this important? Because in later growth stages the living architecture is shaped, which also means that basal stems have to be removed. In order to ensure the water supply of all apical stems, it must be decided when and which stems can be removed. For this decision, the assessment of the morphology, as described in Figure 12, can be of practical help. Therefore, we have conducted morphometric studies focusing on the diameters below and above the inosculation of five tree species. For the selected tree species, *S. alba* and *P.* × *hispanica*, we have compared inosculations with natural branch junctions, by using water flow experiments with a mixture of water and magenta ink to detect the water-conducting tissues in the macroscopic anatomical analyses of transverse sections. From the results of the anatomical studies, we have reconstructed a 3D volumetric visualization of all annual rings and particularly the common annual rings. In order to obtain further detail, we have carried out µ-CT scans of cubed samples from the inosculation and branching regions.

### 4.1. Morphometric Measurements as a Tool for Inosculation Assessment

The scientific questions posed in the introduction are as follows. In the context of practical issues of Baubotanik, we address the question: “Are simple morphometric measurements suitable for assessing whether interconnected stems have merged into a physiological unit with respect to water exchange?”

#### 4.1.1. Theoretical Scenarios of Inosculation Morphology

Initially, we will discuss six theoretical scenarios before interpreting our experimental results within this framework. Figure 12 presents a manually created decision tree [43]. The flowchart-like decision tree originates in the root node at the extreme left (depicted by the arrow) and proceeds via branches over internal nodes (=attribute-based tests; represented by squares) to its leaf nodes (=scenario; represented by triangles). Since this decision tree is a “yes” or “no” classifier and is applicable to all tree species, it seems to be a very appropriate tool for the practice of Baubotanik in the field.

Starting from the root node of the decision tree, we will first focus on the case in which the two diameters below the inosculation do not differ (*Ab* = *Bb*). For the following discussion, we assume that two trees of equivalent diameter have been interconnected. After crosswise or bending connection of Plants A and B, the stems above the inosculation have either developed equally (Scenario I) or unequally (Scenarios II and III). If Plants A and B are exposed to comparable growth conditions in the crown and root area during the course of development, we can assume that their stem diameters below and above the inosculation will develop in approximately the same way. In this case, irrespective of whether the two plants are fused to a physiological unit in terms of water supply, the diameters and diameter ratios do not differ markedly, and the calculated taper ratio *TR* is close to 1.0 (scenario I). However, this does not automatically mean that the inosculation is incomplete, i.e., no physiological connection exists. Another possibility is that the crown and root areas have developed particularly symmetrically, so that no “net water exchange” has occurred, although (partial) inosculation has taken place. Nevertheless, in Scenario I, a complete inosculation is unlikely. If the diameters below the inosculation do not differ significantly, but the diameters above the inosculation are significantly different, then complete inosculation is likely, including water exchange above the individual boundaries of the two plants (Scenario II). Furthermore, if Plant B has a larger diameter above the inosculation than below, complete inosculation is very likely (Scenario III).

If a marked difference between the diameters of Plants A and B can be measured below the inosculation, either the plants were not equivalent regarding their diameters at the time of connection or one of the two interconnected plants was exposed to worse growing conditions, such as a poorer water supply in the root area or the shading of the crown area. However, the difference of the below diameters might not be the result of poor growth conditions (Scenarios IV–VI). An assessment of the growing conditions can be made, for example, by comparing the crown size and leaves of both plants. Further differences of the top diameters (Scenario V) and a larger above diameter than below diameter (Scenario VI) make complete inosculation very likely. In Figure 2b, we see an example of Scenario V in which a complete inosculation is very likely.

In the presented version of the decision tree and with the currently available data, the decision as to whether two diameters differ markedly from each other must be made visually or by diameter assessment by the observer. Our goal is that results from future research will allow us to replace qualitative diameter comparisons with quantitative statements in the form of thresholds that take into account both the tree species and the time period since the stems were conjoined.

#### 4.1.2. Morphology of *Salix alba* and *Platanus* × *hispanica* Inosculations

On the basis of the above theoretical background, we will discuss our results of the diameter measurements in relation to the staining of common annual growth rings in the inosculations. We focus on *S. alba* and *P.* × *hispanica* because their sample size is sufficient for statistical analyses of morphometric data. Since we have found no significant differences in the diameters and the ratios calculated thereof when comparing crosswise and bending connections or fixation with a rope or screw, the data suggest that the connection type and fixation type do not have a marked effect on physiological inosculation, and that we need not distinguish between them (Figure 5a and Supplementary Material Table S1). From the measured diameters below and above the inosculation, we have calculated the diameter ratio of Plants A and B, as a measure of the tree stem taper. In general, plants taper towards the apex in various ways [44]. For both *S. alba* and *P.* × *hispanica*, the measured diameters of Plants A and B below the inosculation do not differ significantly, a result indicating that comparable plants are interconnected and that they have experienced comparable growth conditions and thus developed similarly. Moreover, the measured diameters of Plant A and Plant B above the inosculation are significantly smaller than those below the inosculation, which reflects the taper of the inosculated tree stems. Since the calculated diameter ratios of Plant A (*At/Ab* < 1.0) and Plant B (*Bt/Bb* < 1.0) differ significantly for both species, we can assume that the interconnection caused different developments in the inosculated plants over the period of 8–10 years (Figure 5b). This hypothesis is also supported by the result that the top diameters of Plants A and B of *S. alba* differ significantly (Figure 5d). Based on the pipe model theory [34,35,36,37,38], we can assume that the greater the differences between the upper diameters (*At* and *Bt*) and between the diameter ratios of Plants A and B, the more likely it is that the two plants have become a physiological unit related to water transport. This means that the smaller the calculated taper ratio *TR*, the more unequally Plants A and B have developed after the inosculation, and thus the more likely it is that water exchange has occurred between the two plants in the inosculation region. Interestingly, *S. alba* (Sample #18) with a comparatively high *TR* of 0.91 shows seven common growth rings of the two plants (cf. Figure 6). Thus, we hypothesize that at least all the samples of *S. alba* with a *TR* ≤ 0.91 can exchange water within the inosculation. Notably, the interpretation of a threshold does not allow a reverse conclusion. For example, in the case of *S. alba,* a *TP* value above 0.91 does not necessarily indicate that an inosculation is incomplete, and that no water exchange is possible between plants. Nevertheless, this raises the question of whether this threshold can also be applied to the other tree species, or whether a threshold can be determined for each tree species. Currently, no statement can be made for the other species because of a lack of data. In the case of *P.* × *hispanica* (sample #33) (cf. Figure 7), we see seven common growth rings, but unfortunately, we have been unable to measure the diameters above the inosculations in this sample and, thus, to calculate the diameter ratios or the taper ratio.

We can, therefore, answer the first scientific question by means of the decision tree (Figure 12). First, a strong indication of complete inosculation is provided when, in the same plant, the diameter of one stem above is greater than that below. Second, if the diameters below and above the inosculation do not differ markedly, no statement can be made concerning water exchange from one plant to the other. Moreover, our results showed that following 8–10 years after connection, the practitioner has no need to distinguish between the different types of connections and fixations. Finally, some additional observations can support the decision tree. For example, an experienced arborist can judge whether both plants have developed equally well by comparing crown size and leaves. In addition, the root ball of only one plant can be watered, and the leaves of the other plant can be observed with regard to whether they begin to wilt. The latter would argue against complete inosculation.

### 4.2. Comparison of Artificial Inosculations and Natural Branch Junctions

This brings us to the second scientific question, namely, “What similarities and differences characterize artificial inosculations and natural branch junctions?” With regard to our anatomical study, we can state that the macroscopic observations of the serial cross-sections and the µ-CT scans of inosculations and natural branch junctions are an adequate measure for finding similarities and dissimilarities. The creation of a volumetric 3D reconstruction makes it possible to set up a model for inosculations and branch junctions by abstracting all the details of the individual stems and branches.

#### 4.2.1. Similarities between Inosculations and Branch Junctions

Among the common features, we include the following: single growth rings initially developed in young stems and branches; later, common annular growth rings formed in the inosculations and branch junctions of *S. alba* and *P.* × *hispanica*, respectively. Ink-colored water that passes through the inosculations or branch junctions can identify the peripheral water-conducting tissues, based on their magenta staining. Although we cannot explain the differences in staining between the inosculation and the branch junction of *P.* × *hispanica*, the results nevertheless indicate that, at the point of connection, water transport takes place solely in common growth rings (Figure 6 and Figure 7). The magenta staining in common annual rings is a strong indication that inosculations and natural branching develop comparable structures with respect to water conduction. Marked differences can only be seen in the area directly above and below the inosculation and branch junction and, even here, only in the inner area (approximately 10% to a maximum of 20% of the cross-sectional area). With samples taken from this central zone, the µ-CT scans also document clear differences between branch junctions and inosculations, differences that are much more pronounced in *S. alba* than in *P.* × *hispanica* (Figure 10 and Figure 11). Presumably, the relatively soft wood of *S. alba* is damaged to a greater extent by the joining techniques (crosswise connection and rope fixation) used at the point of connection (cf. Figure 10e,g). However, since these areas are surrounded by extensive regions of common tissue that are far more relevant to water conduction and flexural rigidity, these differences can be considered negligible from a functional perspective at the time of the study. This is different in younger stages, when the two stems are constricted by the ropes or the screws, both of which affect almost the entire area [15].

With regard to the practice of Baubotanik, inosculations and branch junctions can be deduced to be anatomically comparable to a high degree if the common growth rings dominate the structure. Wolff–Vorbeck et al. [45] have shown that the flexural rigidity and the torsional rigidity, both mechanical properties in the linear-elastic range, are largely determined by the cross-sectional patterns of the involved tissues. Since the tissue distributions are remarkably similar for inosculation and branch junction, we can assume that the flexural rigidity and the torsional rigidity are also comparable. This may not be the case when focusing on fracture mechanics [46]. Ultimately, similarities and differences with respect to mechanical properties can only be determined by comparative mechanical investigations of inosculations and branch junctions. However, because of the complex geometry of inosculations and branch junctions, mechanical tests are difficult to analyze. Therefore, Middleton et al. [47] used Finite Element Analysis to develop mechanical models of *S. alba* inosculations that are in good agreement with bending experiments performed in the elastic range. Moreover, the results might also contribute to a better understanding of the natural inosculations of roots [26,27,28]. Vice versa, studies on root systems can be used to verify similarities between branch junctions and inosculations as shown in the present study and to correlate them with mechanical properties (cf. [30]).

The method that we have developed for the volumetric 3D reconstruction of the macroscopic structure has proven to be highly suitable for tracing and comparing the development of natural branch junctions and artificial inosculations (Figure 8 and Figure 9). Although this method is extremely labor intensive and uses destructive examination, it offers the potential of creating models of the internal structures. From our cross-sectional analysis, we know that each stem has individual growth rings in the early phase of inosculation. With the expansion of medullary rays into the cortex, the protuberances from two stems slowly meet. Subsequently, common growth is rapid, increasing the capacity for a water and nutrient exchange between the interconnected tree stems. This finding corresponds well with earlier studies of inosculations, such as the investigation by Millner [32]. Previous studies of xylem development within natural branch junctions have shown a constriction zone attributable to the reduction in the number and width of vessels [48,49].

#### 4.2.2. Dissimilarities between Inosculations and Branch Junctions

Whereas the macroscopic cross-sections of the inosculations and the natural branch junctions are highly similar in many areas, differences become clearer in the digital 3D reconstructions. These differences relate particularly to the first four annual rings *in S. alba* and the first two rings in *P.* × *hispanica*. In the subsequent growth rings, the development becomes increasingly similar. The appearance of the cross-sections is strongly influenced by external factors such as cut-off side branches or the spread of decay. Nevertheless, the important tissue areas can be readily compared. The inner tissues, originally separated and possibly damaged by the connection and fixation technique, are no longer active in terms of water transport. At the time of the investigation, i.e., more than 8–10 years after the trees had been fused, the connection and fixation techniques no longer had any influence on the development of the stems. This hypothesis is supported by the results of the diameter measurements, in which no significant differences have been found for stems of *S. alba* with crosswise or bending connections and for stems of *P.* × *hispanica* fixed with a rope or a screw (Supplementary Materials File S1).

Therefore, we can provide the following answers to our second scientific question. The similarities between inosculations and natural branch junctions are: (1) The wood is notably similar at the point of merging 35 to 85 mm above the point of connection/branching (see Figure 6c,d,g,h and Figure 7c,d,g,h), and the common growth rings are dominant within the area of the connection/branching; (2) Since the cross-sectional tissue pattern is similar, we can assume that the mechanical properties are also comparable; (3) Common water-conducting tissues increase the capacity for water and nutrient exchange. Marked dissimilarities can be found particularly in the central region directly above and below the inosculation and branch junction. In contrast to natural branch junctions, where the cells of the two branches essentially grow alongside each other and can always be assigned to one of the two branches, inosculations result in a highly irregular cell arrangement in which the cells cannot be clearly assigned to one of the two individuals.

### 4.3. Limitations and Opportunities of Conjoined Trees

Overall, the investigated plants showed a comparatively low growth rate in diameter (e.g., approximately 6 mm in diameter after 11 years of growth in *S. alba*) and a rather heterogeneous growth ring development. This can be largely explained by the growing conditions. For about 9 years, the experimental plants were cultivated in relatively small containers and transplanted several times before finally being planted in the ground, and during this period, they were regularly pollarded. In some cases, stem damage from mechanical injury or sunburn also occurred, which affected the entire development of the plants and, thus, for example, the heartwood formation in *S. alba*. Because of the regular and severe pruning and the (associated) injuries, decay occurred in almost all samples, especially in the pioneer tree species, such as *S. alba*, where decay spreads extensively into the wood body. Notably, although our experimental plants have been subjected to the particular growth conditions described above, the resulting morphological, anatomical, and water-conducting consequences are, to some extent, typical for living architecture (i.e., Baubotanik). This is because the plants in living architecture are usually grown under strong competitive conditions, are often heavily pruned, and, when using the technique of plant addition, are sometimes (initially) rooted in containers.

Nevertheless, our studies of the structural and physiological characteristics of trees are a contribution to practitioners who design living architecture by knowing when and where to remove basal stems without compromising the water supply to apical stems (cf. [2]). In addition, our results on trees fused for living architecture make a contribution to future-oriented and sustainable developments of our cities. Leading architects and landscape designers are now meeting the challenges of climate change through urban green infrastructure [1] and nature-based solutions. Creating urban networks of natural spaces is an effective way to participate in sustainable development. In addition to street trees, parks, and urban forests [7,8,9,10,11,12], living trees transformed into artificial architectural structures will play an important role in future cities [2,3,4,5,6]. Scientific long-term studies of the development of inosculated trees, such as the one presented here, are thus urgently needed for this idea to be brought about and safely implemented in urban and rural areas.

## 5. Conclusions

In the context of climate change, new forms of urban greenery and green architecture are being explored to provide ecosystem services in dense urban areas. One particularly promising, but so far only occasionally realized approach, is the inosculation of trees as a basis for artificial living structures. Living architecture can thus contribute to urban networks of natural spaces providing nature-based solutions in the form of urban green infrastructure. A reliable, comprehensive, and simple evaluation method of the development status of inosculations is required for the realization and safe use of artificial living structures in urban areas. When designing living architecture, practitioners should know when and where to remove basal stems without compromising the water supply to apical stems. The study presented here makes a suitable contribution to this evaluation by providing a decision tree and some rules of thumb for assessing the developmental status of inosculated stems. Our approach is based on measurements of the diameters of the conjoined stems above and below the inosculation and can be applied by an experienced arborist directly in the field in a similar way to established assessments within the field of arboriculture [50,51]. Through simple measurements or even mere visual assessments, the degree of physiological connectivity of single inosculations or, on a larger scale, more complex structures with multiple inosculations can be quickly evaluated to make immediate on-site maintenance decisions. In our comparative study, we have found similarities that allow the morphological, anatomical, physiological, and mechanical insights and modeling approaches available for natural branch junctions to be applied to inosculations. Once this is confirmed by further research, the extremely time-consuming and lengthy process of cultivating inosculations for experimental purposes can be, at least partially, bypassed. In addition, implementation projects on a larger scale can be initiated much more quickly so that living architecture can rapidly contribute directly to human well-being and can demonstrate its potential for adapting our cities to climate change. The presented decision tree is highly suitable for the assessment of whether conjoined plants can supply each other with water within living architecture. We plan further to strengthen the qualitative criteria (i.e., smaller than, larger than) of the decision tree presented here in future research in order to allow the specification of quantitative criteria, together with indications of thresholds, related to appropriate tree species and durations of inosculation.

## Figures and Tables

**Table 1:** Statistical analyses of *Platanus* × *hispanica* (*N* = 8) and *Salix alba* (*N* = 17). Variables are given as median and interquartile range in round brackets.

Variable	*Platanus* × *hispanica*	*Salix alba*
*At* [cm]	5.7 (1.5)	7.4 (1.1)
*Ab* [cm]	7.5 (2.5)	9.8 (0.6)
*Bt* [cm]	6.0 (1.9)	8.2 (1.8)
*Bb* [cm]	6.6 (2.9)	9.2 (1.8)
*At/Ab* [-]	0.80 (0.07)	0.78 (0.06)
*Bt/Bb* [-]	0.85 (0.13)	0.88 (0.06)
*(At/Ab)/(Bt/Bb)* [-]	0.92 (0.06)	0.87 (0.09)

## Data Availability

The data presented in this study are available in the text, tables, figures and Supplementary Materials.

## Supplementary Materials

The following supporting information can be downloaded at: https://www.mdpi.com/article/10.3390/plants12061385/s1, Table S1: Data table of morphometric studies, File S1: Statistical analyses of morphometric studies, Video S1: Serial sections of an inosculation and a branch junction of *S. alba*., Video S2: Serial sections of an inosculation and a branch junction of *P.* × *hispanica*, Video S3: Simulation of the development of an inosculation of *S. alba*, Video S4: Simulation of the development of a natural branch junction of *S. alba,* Video S5: Simulation of the development of an inosculation of *P.* × *hispanica*, Video S6: Simulation of the development of a natural branch junction of *P.* × *hispanica*, Videos S7a–d: Videos of the µ-CT scans of *S. alba,* Videos S8a–d: Videos of the µ-CT scans of *P.* × *hispanica*.

## References

[1] United Nations (2015). Transforming Our World: The 2030 Agenda for Sustainable Development: A/RES/70/1. https://www.un.org/en/development/desa/population/migration/generalassembly/docs/globalcompact/A_RES_70_1_E.pdf.

[2] Ludwig F., Löschke S. (2016). Baubotanik: Designing with Living Material. Materiality and Architecture.

[3] Kirsch K. (1996). Naturbauten aus Lebenden Gehölzen.

[4] Arbona J., Greden L., Joachim M. (2003). Nature’s Technology: The Fab Tree Hab House. Thresholds.

[5] Reams R. (2005). Arborsculpture. Solutions for a Small Planet.

[6] Wang X., Gard W., Borska H., Ursem B., van de Kuilen J. (2020). Vertical greenery systems: From plants to trees with self-growing interconnections. Eur. J. Wood Wood Prod..

[7] Bowler D.E., Buyung-Ali L., Knight T.M., Pullin A.S. (2010). Urban greening to cool towns and cities: A systematic review of the empirical evidence. Landsc. Urban Plan..

[8] Nowak D.J., Crane D.E. (2002). Carbon storage and sequestration by urban trees in the USA. Environ. Pollut..

[9] Kuehler E., Hathaway J., Tirpak A. (2017). Quantifying the benefits of urban forest systems as a component of the green infrastructure stormwater treatment network. Ecohydrology.

[10] Rötzer T., Rahman M.A., Moser-Reischl A., Pauleit S., Pretzsch H. (2019). Process based simulation of tree growth and ecosystem services of urban trees under present and future climate conditions. Sci. Total Environ..

[11] Rahman M.A., Stratopoulos L.M.F., Moser-Reischl A., Zölch T., Häberle K.-H., Rötzer T., Pretzsch H., Pauleit S. (2020). Traits of trees for cooling urban heat islands: A meta-analysis. Build. Environ..

[12] Ludwig F., Schönle D., Bellers M. (2015). Klimaaktive baubotanische Stadtquartiere, Bautypologien und Infrastrukturen: Modellprojekte und Planungswerkzeuge. Klimopass-Berichte. LUBW Landesanstalt für Umwelt, Messungen und Naturschutz Baden-Württemberg. https://pd.lubw.de/91679.

[13] Ludwig F., De Bruyn G., Ludwig F., Schwertfeger H. (2009). Baubotanik—Lebendarchitektur. Lebende Bauten−Trainierbare Tragwerke.

[14] Ludwig F., Baier B., Koenen R., Müller J., Schmerbach S. (2008). Baubotanik—Möglichkeiten und Grenzen des Konstruierens lebender Tragwerke. Konstruktion und Gestalt—Leichte Konstruktionen.

[15] Ludwig F. (2012). Botanische Grundlagen der Baubotanik und deren Anwendung im Entwurf. Ph.D. Thesis.

[16] Ludwig F., Middleton W., Vees U. (2019). Baubotanik: Living Wood and Organic Joints. Rethinking Wood—Future Dimensions of Timber Assembly.

[17] Ludwig F., Middleton W., Gallenmüller F., Rogers P., Speck T. (2019). Living bridges using aerial roots of *Ficus elastica*—An interdisciplinary perspective. Sci. Rep..

[18] Middleton W., Habibi A., Shankar S., Ludwig F. (2020). Characterizing Regenerative Aspects of Living Root Bridges. Sustainability.

[19] Graefe R. (1987). Geleitete Linden. Daidalos Z. Für Archit. Kunst Kult..

[20] Graefe R. (2014). Bauten aus Lebenden Bäumen.

[21] Ludwig F. (2018). Lebende Konstruktionen—Eine historische Einführung in die Baubotanik. nodium.

[22] Cattle C. (2010). Grown furniture: A move towards design for sustainability. Ph.D. Thesis.

[23] Harris R.W. (1992). Arboriculture: Integrated Management of Landscape Trees, Shrubs, and Vines.

[24] Roloff A. (2013). Baumpflege.

[25] Wang X., Gard W., de Vries N., van de Kuilen J.-W. (2022). Anatomical and Mechanical Features of Self-Growing Connections in Plants. preprint.

[26] Graham B., Bormann F. (1966). Natural root grafts. Bot. Rev..

[27] Bormann F. (1966). The structure, function, and ecological significance of root grafts in *Pinus strobus* L. Ecol. Monogr..

[28] Tarroux E., DesRochers A., Tremblay F. (2014). Molecular analysis of natural root grafting in jack pine (*Pinus banksiana*) trees: How does genetic proximity influence anastomosis occurrence?. Tree Genet. Genomes.

[29] Tarroux E., DesRochers A. (2011). Effect of natural root grafting on growth response of jack pine (*Pinus banksiana*; Pinaceae). Am. J. Bot..

[30] Basnet K., Scatena F., Likens G.E., Lugo A.E. (1993). Ecological consequences of root grafting in tabonuco (*Dacryodes excelsa*) trees in the Luquillo Experimental Forest, Puerto Rico. Biotropica.

[31] Bormann F., Graham B.F. (1959). The occurrence of natural root grafting in eastern white pine, *Pinus strobus* L., and its ecological implications. Ecology.

[32] Millner E.M. (1932). Natural grafting in *Hedera helix*. New Phytol..

[33] Harrington M.J., Speck O., Speck T., Wagner S., Weinkamer R., Hager M., van der Zwaag S., Schubert U. (2015). Biological archetypes for self-healing materials. Self-Healing Materials. Advances in Polymer Science.

[34] Shinozaki K., Yoda K., Hozumi K., Kira T. (1964). A quantitative Analysis of Plant Form—The Pipe Model Theory: I. Basic Analyses. Jpn. J. Ecol..

[35] Shinozaki K., Yoda K., Hozumi K., Kira T. (1964). A quantitative Analysis of Plant Form—The Pipe Model Theory: II. Further Evidence of the Theory and its Application in Forest Ecology. Jpn. J. Ecol..

[36] Lehnebach R., Beyer R., Letort V., Heuret P. (2018). The pipe model theory half a century on: A review. Ann. Bot..

[37] Godin C. (2000). Representing and encoding plant architecture: A review. Ann. For. Sci..

[38] Perttunen J., Änen R.S., Nikinmaa E., Salminen H., Saarenmaa H. (1996). LIGNUM: A tree model based on simple structural units. Ann. Bot..

[39] Mattheck C., Kubler H. (1997). Wood—The internal Optimization of Trees.

[40] Burkhart H.E., Tomé M. (2012). Tree form and stem taper. Modeling Forest Trees and Stands.

[41] Veit N. (2012). Wie Verhalten sich Verwachsungen bei Unterschiedlichen Verbindungstechniken. Tech. Staatsschule Für Gart. Hohenh..

[42] R Core Team (2019). R: A Language and Environment for Statistical Computing.

[43] Kingsford C., Salzberg S.L. (2008). What are decision trees?. Nat. Biotechnol..

[44] Langer M., Kelbel M.C., Speck T., Müller C., Speck O. (2021). Twist-to-bend ratios and safety factors of petioles having various geometries, sizes and shapes. Front. Plant Sci..

[45] Wolff-Vorbeck S., Speck O., Langer M., Speck T., Dondl P.W. (2022). Charting the twist-to-bend ratio of plant axes. J. R. Soc. Interface.

[46] Speck T., Spatz H.-C., Vogellehner D. (1990). Contributions to the Biomechanics of Plants. I. Stabilities of Plant Stems with Strengthening Elements of Different Cross-Sections against Weight and Wind Forces. Bot. Acta.

[47] Middleton W., Erdal H.I., Detter A., D’Acunto P., Ludwig F. (2023). Comparing structural models of linear elastic responses to bending in inosculated joints. Trees.

[48] Isebrands J.G., Larson P.R. (1977). Vascular anatomy of the nodal region in *Populus deltoides* Bartr. Am. J. Bot..

[49] Larson P.R., Isebrands J.G. (1978). Functional significance of the nodal constricted zone in *Populus deltoides*. Can. J. Bot..

[50] Li H., Zhang X., Li Z., Wen J., Tan X. (2022). A review of research on tree risk assessment methods. Forests.

[51] Linhares C.S., Gonçalves R., Martins L.M., Knapic S. (2021). Structural stability of urban trees using visual and instrumental techniques: A review. Forests.

