# Supplementary material for: Conjoining Trees for the Provision of Living Architecture in Future Cities: A Long-Term Inosculation Study
Source: Plants (Basel). 2023 Mar 20;12(6):1385. doi: 10.3390/plants12061385 (PMC10058916; doi:10.3390/plants12061385)
Supplement: Supplementary file 1 [file plants-12-01385-s001.zip › S01.pdf]

Conjoining Trees for the Provision of Living Architecture in Future Cities: a Long-Term Inosculation Study

Max D. Mylo [1,2,3,+], Ferdinand Ludwig [4,+], Mohammad Asrafur Rahman [5], Qiguan Shu [4], Christoph Fleckenstein [4], Thomas Speck [1,2] and Olga Speck [1,2,*]

1 Plant Biomechanics Group @ Botanic Garden Freiburg, University of Freiburg, D-79104 Freiburg, Germany; max.mylo@livmats.uni-freiburg.de (M.M.) thomas.speck@biologie.uni-freiburg.de (T.S.); olga.speck@biologie.uni-freiburg.de (O.S.)
2 Cluster of Excellence *liv*MatS @ FIT—Freiburg Center for Interactive Materials and Bioinspired Technologies, University of Freiburg, D-79110 Freiburg, Germany; max.mylo@livmats.uni-freiburg.de (M.M.) thomas.speck@biologie.uni-freiburg.de (T.S.); olga.speck@biologie.uni-freiburg.de (O.S.)
3 Fraunhofer Institute for Mechanics of Materials IWM, D-79108 Freiburg, Germany; max.mylo@livmats.uni-freiburg.de (M.M.)
4 Green Technologies in Landscape Architecture, School of Engineering and Design, Research Group Baubotanik, Technical University of Munich, D-80333 Munich, Germany; ferdinand.ludwig@tum.de (F.L.), christoph.fleckenstein@tum.de (C.F.), qiguan.shu@tum.de (Q.S.)
5 Strategic Landscape Planning and Management, School of Life Sciences, Weihenstephan, Technical University of Munich, D-85354 Freising, Germany; ma.rahman@tum.de (M.A.R.)
+ shared first authorship
* Correspondence: olga.speck@biologie.uni-freiburg.de

Supplementary Materials S01:

Data Table of Morphometric Studies

#	tree species	connection	fixa-tion	*At* [cm]	*Ab* [cm]	*Bt* [cm]	*Bb* [cm]	*At/Ab* [–]	*Bt/Bb* [–]	*(At/Ab)/(Bt/Bb)* [–]	Morpho-metrics	µ-CT	3D
1	*Acer platanoides*	crosswise	rope	6.3	8.2	6.3	7.7	0.77	0.82	0.939	+		
2	*Acer platanoides*	crosswise	rope	6.1	7.7	7.8	9.5	0.79	0.82	0.965	+		
3	*Alnus glutinosa*	crosswise	rope	7.6	11.4	11.9	16.3	0.67	0.73	0.913	+		
4	*Betula pendula*	crosswise	rope	4.4	6.3	5.3	5.3	0.70	1.00	0.698	+		
5	*Betula pendula*	crosswise	rope	4.0	6.2	5.6	6.6	0.65	0.85	0.760	+		
6	*Betula pendula*	crosswise	rope	6.6	9.1	7.5	8.5	0.73	0.88	0.822	+		
7	*Betula pendula*	crosswise	rope	7.1	9.4	6.3	8.0	0.76	0.79	0.959	+		
8	*Platanus* x *hispanica*	bending	rope	7.1	8.4	6.2	6.5	0.85	0.95	0.886	+		
9	*Platanus* x *hispanica*	crosswise	rope	6.0	8.0	6.6	8.2	0.75	0.80	0.932	+		
10	*Platanus* x *hispanica*	crosswise	rope	5.7	6.9	5.8	6.6	0.83	0.88	0.940	+		
11	*Platanus* x *hispanica*	crosswise	screw	5.6	8.2	6.5	8.1	0.68	0.80	0.851	+		
12	*Platanus* x *hispanica*	crosswise	screw	4.6	5.6	4.8	5.1	0.82	0.94	0.873	+		
13	*Platanus* x *hispanica*	crosswise	screw	4.6	5.4	4.1	4.4	0.85	0.93	0.914	+		
14	*Platanus* x *hispanica*	crosswise	screw	6.3	8.0	7.0	8.5	0.79	0.82	0.956	+	+	
15	*Platanus* x *hispanica*	crosswise	screw	3.9	5.1	4.2	5.3	0.76	0.79	0.965	+		
16	*Salix alba*	bending	rope	7.0	9.0	8.2	7.9	0.78	1.04	0.749	+		
17	*Salix alba*	bending	rope	7.7	9.9	8.5	9.0	0.78	0.94	0.824	+		
18	*Salix alba*	bending	rope	7.0	9.6	6.7	8.4	0.73	0.80	0.914	+		+
19	*Salix alba*	bending	rope	10.2	12.8	10.2	12.1	0.80	0.84	0.945	+		
20	*Salix alba*	bending	rope	8.2	10.1	7.2	8.5	0.81	0.85	0.958	+		
21	*Salix alba*	crosswise	rope	4.9	7.4	5.1	5.1	0.66	1.00	0.662	+		
22	*Salix alba*	crosswise	rope	7.5	10.1	9.3	9.2	0.74	1.01	0.735	+		
23	*Salix alba*	crosswise	rope	7.4	10.4	10.7	11.6	0.71	0.92	0.771	+		
24	*Salix alba*	crosswise	rope	6.4	10.0	6.5	8.4	0.64	0.77	0.827	+		
25	*Salix alba*	crosswise	rope	7.4	9.8	8.2	9.3	0.76	0.88	0.856	+	+	
26	*Salix alba*	crosswise	rope	7.1	9.6	8.0	9.3	0.74	0.86	0.860	+		
27	*Salix alba*	crosswise	rope	6.9	8.8	9.2	10.2	0.78	0.90	0.869	+		
28	*Salix alba*	crosswise	rope	8.4	11.1	9.8	11.3	0.76	0.87	0.873	+		
29	*Salix alba*	crosswise	rope	7.2	9.1	7.5	8.4	0.79	0.89	0.886	+		
30	*Salix alba*	crosswise	rope	10.2	12.2	8.1	8.8	0.84	0.92	0.908	+		
31	*Salix alba*	crosswise	rope	8.0	9.5	9.9	11.2	0.84	0.88	0.953	+		
32	*Salix alba*	crosswise	rope	8.1	9.7	8.6	9.9	0.84	0.87	0.961	+		
33	*Platanus* x *hispanica*	crosswise	screw	—	5.1	—	5.2						+

#	height t [cm]	height b [cm]	joint height [cm]
1	35	10	24
2	35	10	34
3	35	10	30
4	35	10	21
5	35	10	20
6	35	10	30
7	**20**	10	24
8	35	10	24
9	35	10	36
10	35	10	24
11	35	**5**	34
12	35	10	43
13	35	10	49
14	35	10	37
15	35	10	57
16	35	10	40
17	35	10	56
18	35	10	49
19	35	10	40
20	35	10	37
21	35	10	44
22	35	10	22
23	35	10	46
24	35	10	47
25	35	10	42
26	35	10	37
27	35	10	42
28	35	10	42
29	35	10	46
30	35	10	41
31	35	10	26
32	35	**5**	25
33	35	10	49
