# Supplementary material for: Conjoining Trees for the Provision of Living Architecture in Future Cities: A Long-Term Inosculation Study
Source: Plants (Basel). 2023 Mar 20;12(6):1385. doi: 10.3390/plants12061385 (PMC10058916; doi:10.3390/plants12061385)
Supplement: Supplementary file 1 [file plants-12-01385-s001.zip › S02.pdf]

Conjoining Trees for the Provision of Living Architecture in Future Cities: a Long-Term Inosculation Study

Max D. Mylo [1,2,3,+], Ferdinand Ludwig [4,+], Mohammad Asrafur Rahman [5], Qiguan Shu [4], Christoph Fleckenstein [4], Thomas Speck [1,2] and Olga Speck [1,2,*]

1 Plant Biomechanics Group @ Botanic Garden Freiburg, University of Freiburg, D-79104 Freiburg, Germany; max.mylo@livmats.uni-freiburg.de (M.M.) thomas.speck@biologie.uni-freiburg.de (T.S.); olga.speck@biologie.uni-freiburg.de (O.S.)
2 Cluster of Excellence *liv*MatS @ FIT—Freiburg Center for Interactive Materials and Bioinspired Technologies, University of Freiburg, D-79110 Freiburg, Germany; max.mylo@livmats.uni-freiburg.de (M.M.) thomas.speck@biologie.uni-freiburg.de (T.S.); olga.speck@biologie.uni-freiburg.de (O.S.)
3 Fraunhofer Institute for Mechanics of Materials IWM, D-79108 Freiburg, Germany; max.mylo@livmats.uni-freiburg.de (M.M.)
4 Green Technologies in Landscape Architecture, School of Engineering and Design, Research Group Baubotanik, Technical University of Munich, D-80333 Munich, Germany; ferdinand.ludwig@tum.de (F.L.), christoph.fleckenstein@tum.de (C.F.), qiguan.shu@tum.de (Q.S.)
5 Strategic Landscape Planning and Management, School of Life Sciences, Weihenstephan, Technical University of Munich, D-85354 Freising, Germany; ma.rahman@tum.de (M.A.R.)
+ shared first authorship
* Correspondence: olga.speck@biologie.uni-freiburg.de

Supplementary Materials S02: Statistical analyses

R version 4.0.4 (2021-02-15)

1. *Platanus* x *hispanica*

Shapiro-Wilk normality test
data: trees[8:15, "**At**"]
W = 0.9673, p-value = 0.876

Shapiro-Wilk normality test
data: trees[8:15, "**Ab**"]
W = 0.83739, p-value = 0.0708

Shapiro-Wilk normality test
data: trees[8:15, "**Bt**"]
W = 0.89245, p-value = 0.2466

Shapiro-Wilk normality test
data: trees[8:15, "**Bb**"]
W = 0.90999, p-value = 0.354

Levene's test

											Varianzquotient					
			At [cm]	Ab [cm]	Bt [cm]	Bb [cm]	At/Ab [-]	Bt/Bb [-]		kritischer Wert	Ab - At	Bb - Bb	At/Ab - Bt/Bb	Ergebnis: Varianzquatient < kritischer Wert --> gleiche varianzen		
Platanus x *hispanica*	Varianzanalyse		1.10	1.93	1.29	2.46	0.00327	0.00469		3.79	1.76	1.91	1.44	gleiche Varianzen		

Paired t-test
data: trees[8:15, "**At**"] and trees[8:15, "**Ab**"]
t = -7.0447, df = 7, p-value = 0.0002033
alternative hypothesis: true difference in means is not equal to 0
95 percent confidence interval:
 -1.9701011 -0.9798989
sample estimates:
mean of the differences
 -1.475

Paired t-test
data: trees[8:15, "**Bt**"] and trees[8:15, "**Bb**"]
t = -4.4741, df = 7, p-value = 0.002887
alternative hypothesis: true difference in means is not equal to 0
95 percent confidence interval:
 -1.4329791 -0.4420209
sample estimates:
mean of the differences
 -0.9375

Paired t-test
data: trees[8:15, "**At**"] and trees[8:15, "**Bt**"]
t = -0.80983, df = 7, p-value = 0.4447
alternative hypothesis: true difference in means is not equal to 0
95 percent confidence interval:
 -0.6859801 0.3359801
sample estimates:
mean of the differences
 -0.175

Paired t-test
data: trees[8:15, "**Ab**"] and trees[8:15, "**Bb**"]
t = 1.3176, df = 7, p-value = 0.2291
alternative hypothesis: true difference in means is not equal to 0
95 percent confidence interval:
 -0.2880595 1.0130595
sample estimates:
mean of the differences
 0.3625

Shapiro-Wilk normality test
data: trees[8:15, "**At.Ab**"]
W = 0.90727, p-value = 0.3352

Shapiro-Wilk normality test
data: trees[8:15, "**Bt.Bb**"]
W = 0.83777, p-value = 0.07145

Paired t-test
data: trees[16:32, "**At.Ab**"] and trees[16:32, "**Bt.Bb**"]
t = -6.0307, df = 16, p-value = 1.748e-05
alternative hypothesis: true difference in means is not equal to 0
95 percent confidence interval:
 -0.17808252 -0.08544689

sample estimates:
mean of the differences
-0.1317647

Paired t-test
data: trees[8:15, "**At.Ab**"] and trees[8:15, "**Bt.Bb**"]
t = -5.439, df = 7, p-value = 0.0009673
alternative hypothesis: true difference in means is not equal to 0
95 percent confidence interval:
-0.10401953 -0.04098047
sample estimates:
mean of the differences
-0.0725

At rope - screw: Welch Two Sample t-test
data: trees[9:10, "At"] and trees[11:15, "At"]
t = 1.8936, df = 4.7675, p-value = 0.1196
alternative hypothesis: true difference in means is not equal to 0
95 percent confidence interval:
-0.3210365 2.0210365
sample estimates:
mean of x mean of y
5.85 5.00

Ab rope - screw: Welch Two Sample t-test
data: trees[9:10, "Ab"] and trees[11:15, "Ab"]
t = 1.137, df = 4.0078, p-value = 0.3189
alternative hypothesis: true difference in means is not equal to 0
95 percent confidence interval:
-1.425566 3.405566
sample estimates:
mean of x mean of y
7.45 6.46

Bt rope - screw: Welch Two Sample t-test
data: trees[9:10, "Bt"] and trees[11:15, "Bt"]
t = 1.2187, df = 4.6669, p-value = 0.281
alternative hypothesis: true difference in means is not equal to 0
95 percent confidence interval:
-1.016733 2.776733
sample estimates:
mean of x mean of y
6.20 5.32

Bb rope - screw: Welch Two Sample t-test
data: trees[9:10, "Bb"] and trees[11:15, "Bb"]
t = 0.96523, df = 3.3925, p-value = 0.3981
alternative hypothesis: true difference in means is not equal to 0
95 percent confidence interval:
-2.342411 4.582411
sample estimates:
mean of x mean of y
7.40 6.28

At/Ab rope - screw: Welch Two Sample t-test
data: trees[9:10, "At.Ab"] and trees[11:15, "At.Ab"]
t = 0.20203, df = 2.1902, p-value = 0.8571
alternative hypothesis: true difference in means is not equal to 0

95 percent confidence interval:
 -0.1861935 0.2061935
sample estimates:
mean of x mean of y
    0.79   0.78

Bt/Bb rope - screw: Welch Two Sample t-test
data: trees[9:10, "Bt.Bb"] and trees[11:15, "Bt.Bb"]
t = -0.30988, df = 2.4991, p-value = 0.7806
alternative hypothesis: true difference in means is not equal to 0
95 percent confidence interval:
 -0.2006209 0.1686209
sample estimates:
mean of x mean of y
   0.840   0.856

TR rope - screw: Welch Two Sample t-test
data: trees[9:10, 11] and trees[11:15, 11]
t = 0.9477, df = 4.3285, p-value = 0.3931
alternative hypothesis: true difference in means is not equal to 0
95 percent confidence interval:
 -0.04241305 0.08841305
sample estimates:
mean of x mean of y
   0.935   0.912

2. *Salix alba*

Shapiro-Wilk normality test
data: trees[16:32, "**At**"]
W = 0.9098, p-value = 0.09925

Shapiro-Wilk normality test
data: trees[16:32, "**Ab**"]
W = 0.91975, p-value = 0.1462

Shapiro-Wilk normality test
data: trees[16:32, "**Bt**"]
W = 0.97745, p-value = 0.9304

Shapiro-Wilk normality test
data: trees[16:32, "**Bb**"]
W = 0.92857, p-value = 0.2059

Shapiro-Wilk normality test
data: trees[16:32, "**At.Ab**"]
W = 0.93609, p-value = 0.2747

Shapiro-Wilk normality test
data: trees[16:32, "**Bt.Bb**"]
W = 0.95606, p-value = 0.559

Levene's test

										Varianzquotient			
		At [cm]	Ab [cm]	Bt [cm]	Bb [cm]	At/Ab [-]	Bt/Bb [-]		kritischer Wert	Ab - At	Bb - Bb	At/Ab - Bt/Bb	Ergebnis: Varianzquatient < kritischer Wert --> gleiche varianzen
Salix alba	Varianzanalyse	1.59	1.55	2.13	2.81	0.00326	0.00505		2.33	0.98	1.32	1.55	gleiche Varianzen

Paired t-test
data: trees[16:32, "**At**"] and trees[16:32, "**Ab**"]
t = -18.083, df = 16, p-value = 4.496e-12
alternative hypothesis: true difference in means is not equal to 0
95 percent confidence interval:
 -2.595915 -2.051144
sample estimates:
mean of the differences
 -2.323529

Paired t-test
data: trees[16:32, "**Bt**"] and trees[16:32, "**Bb**"]
t = -6.2016, df = 16, p-value = 1.269e-05
alternative hypothesis: true difference in means is not equal to 0
95 percent confidence interval:
 -1.333940 -0.654295
sample estimates:
mean of the differences
 -0.9941176

Paired t-test
data: trees[16:32, "**At**"] and trees[16:32, "**Bt**"]
t = -2.3112, df = 16, p-value = 0.03448
alternative hypothesis: true difference in means is not equal to 0
95 percent confidence interval:
 -1.36462771 -0.05890171
sample estimates:
mean of the differences
 -0.7117647

Paired t-test
data: trees[16:32, "**Ab**"] and trees[16:32, "**Bb**"]
t = 1.9444, df = 16, p-value = 0.06964
alternative hypothesis: true difference in means is not equal to 0
95 percent confidence interval:
 -0.05573462 1.29102874
sample estimates:
mean of the differences
 0.6176471

At bending - crosswise: Welch Two Sample t-test
data: trees[16:20, "At"] and trees[21:32, "At"]
t = 0.81122, df = 7.2027, p-value = 0.4432
alternative hypothesis: true difference in means is not equal to 0
95 percent confidence interval:
 -1.066238 2.189571
sample estimates:
mean of x mean of y
 8.020000 7.458333

Ab bending - crosswise: Welch Two Sample t-test
data: trees[16:20, "Ab"] and trees[21:32, "Ab"]
t = 0.63743, df = 6.278, p-value = 0.5464
alternative hypothesis: true difference in means is not equal to 0
95 percent confidence interval:

-1.319675 2.263009
sample estimates:
mean of x mean of y
10.280000 9.808333

Bt bending - crosswise: Welch Two Sample t-test
data: trees[16:20, "Bt"] and trees[21:32, "Bt"]
t = -0.32949, df = 8.6533, p-value = 0.7496
alternative hypothesis: true difference in means is not equal to 0
95 percent confidence interval:
-1.963744 1.467077
sample estimates:
mean of x mean of y
8.160000 8.408333

Bb bending - crosswise: Welch Two Sample t-test
data: trees[16:20, "Bb"] and trees[21:32, "Bb"]
t = -0.23416, df = 7.8358, p-value = 0.8209
alternative hypothesis: true difference in means is not equal to 0
95 percent confidence interval:
-2.303781 1.880447
sample estimates:
mean of x mean of y
9.180000 9.391667

At/Bb bending - crosswise: Welch Two Sample t-test
data: trees[16:20, "At.Ab"] and trees[21:32, "At.Ab"]
t = 0.91978, df = 14.573, p-value = 0.3727
alternative hypothesis: true difference in means is not equal to 0
95 percent confidence interval:
-0.02867097 0.07200430
sample estimates:
mean of x mean of y
0.7800000 0.7583333

Bt/Bb bending - crosswise: Welch Two Sample t-test
data: trees[16:20, "Bt.Bb"] and trees[21:32, "Bt.Bb"]
t = -0.074799, df = 5.5022, p-value = 0.943
alternative hypothesis: true difference in means is not equal to 0
95 percent confidence interval:
-0.1205555 0.1135555
sample estimates:
mean of x mean of y
0.8940   0.8975

TR rope - screw: Welch Two Sample t-test
data: trees[16:20, 11] and trees[21:32, 11]
t = 0.64094, df = 7.2878, p-value = 0.5412
alternative hypothesis: true difference in means is not equal to 0
95 percent confidence interval:
-0.08112992 0.14212992
sample estimates:
mean of x mean of y
0.8780   0.8475
